# Molecular and Functional Characterization of the Odorant Receptor2 (OR2) in the Tiger Mosquito *Aedes albopictus*

**DOI:** 10.1371/journal.pone.0036538

**Published:** 2012-05-14

**Authors:** Filippo Scialò, Bill S. Hansson, Ennio Giordano, Catello L. Polito, F. Anna Digilio

**Affiliations:** 1 Institute of Genetics and Biophysics A. Buzzati-Traverso, CNR, Naples, Italy; 2 Evolutionary Neuroethology, Max Planck Institute for Chemical Ecology Jena, Germany; 3 Department of Biological Sciences, University of Naples Federico II, Naples, Italy; Barnard College, Columbia University, United States of America

## Abstract

In mosquitoes, the olfactory system plays a crucial role in many types of behavior, including nectar feeding, host preference selection and oviposition. *Aedes albopictus*, known also as the tiger mosquito, is an anthropophilic species, which in the last few years, due to its strong ecological plasticity, has spread throughout the world. Although long considered only a secondary vector of viruses, the potential of its vector capacity may constitute a threat to public health. Based on the idea that an improved understanding of the olfactory system of mosquitoes may assist in the development of control methods that interfere with their behavior, we have undertaken a study aimed at characterizing the *A. albopictus* Odorant Receptors. Here we report the identification, cloning and functional characterization of the *Aal*OR2 ortholog, that represents the first candidate member of the odorant receptor (OR) family of proteins from *A. albopictus*. AalOR2 is expressed in the larval heads and antennae of adults. Our data indicate that *A. albopictus* OR2 (AalOR2) shares a high degree of identity with other mosquito OR2 orthologs characterized to date, confirming that OR2 is one of the most conserved mosquito ORs. Our data indicate that AalOR2 is narrowly tuned to indole, and inhibited by (-)-menthone. In agreement with this results, these two compounds elicit two opposite effects on the olfactory-based behavior of *A. albopictus* larvae, as determined through a larval behavioral assay. In summary, this work has led to the cloning and de-orphaning of the first Odorant Receptor in the tiger mosquito *A. albopictus*. In future control strategies this receptor may be used as a potential molecular target.

## Introduction

*A. albopictus* (Skuse) (Diptera: Culicidae), also known as the tiger mosquito, is one of the most widespread mosquito species in the world. Originally, it was present only in the tropical forests of South-East Asia but based on its strong ecological plasticity and on the worldwide commerce in used tires [1], it has been able to colonize most of the world. Although the species is not a major vector for the most devastating diseases, its vector capacity raises concerns and is the reason for a public health alert. Recent reports indeed show that *A. albopictus* is an epidemic vector of the dengue and chikungunya arboviruses in most of the islands in the Indian Ocean, where the mosquito *A. aegypti*, usually implicated in such outbreaks, is virtually absent [2]. Also in Italy Calzolari et al [3], and Dutto and Bertero [4], have connected the presence of this exotic mosquito to a health risk, supporting the ability of *A. albopictus* to serve as a bridge vector, capable of mediating the spillover of a virus from a rural to an urban cycle. Comprehensive behavioral studies have indicated that the most crucial cues regulating many activities of mosquitoes, such as host-seeking, searching for oviposition sites and feeding, are volatiles emitted from hosts or plants [5], [6]. The ability of mosquitoes to identify a host for a blood meal or a correct site where to lay eggs via olfactory cues is conferred by a rich repertoire of Odorant Receptors (ORs) expressed in olfactory sensory neurons (OSNs) housed in the olfactory sensilla. Insect ORs belong to the 7-transmembrane type, but show no homology to any other ORs identified in vertebrates or nematodes. They also display an inverted insertion into the membrane [7], [8]. It has been shown that insect ORs function as heteromeric ligand-gated ion channels [9], [10], [11], consisting of an olfactory receptor and a highly conserved member of this family (the olfactory co-receptor *Orco*), which is not directly involved in the recognition of odor molecules but in the transduction cascade [12], [13].

In the last decade genomes of many species have been released, allowing the identification of large OR families. In mosquitoes these genomic analyses have led to the identification of 79 ORs in the malaria vector mosquito *A. gambiae* [14], 131 in the yellow fever and dengue virus vector *A. aegypti* [15], and 180 in *C. pipiens quienquiefasciatus* [16]. The genome of *A. albopictus* is, however, so far uncharacterized, and to date no ORs have been identified. A further significant insight into the mosquito sense of smell has recently been obtained by the functional characterization of fifty Ors in *A. gambiae*. The deorphanization was performed using both two-electrode, voltage-clamp electrophysiology in *Xenopus* oocytes [17] and the *D. melanogaster* “empty neuron” system [18]. In particular, the results obtained by Carey and colleagues [18] indicate that *A. gambiae*, like *D. melanogaster*, uses a combination of both narrowly tuned (specialist) and broad spectrum (generalist) ORs, and that each AgOR has a distinct odor-response profile and tuning breadth; moreover, certain odors activate some OSN types while they inhibit others, suggesting that responses to odors could be integrated already at the antennal level. In mosquitoes, one narrowly tuned receptor that has been studied very well is OR2. *A. gambiae* OR2 (AgOr2) is tuned to a small set of aromatics including indole [18], which is an oviposition attractant for *C. pipiens* and has been found to constitute nearly 30% of the volatile headspace of human sweat [19]. As shown by Xia and collaborators [20], AgOR2 is expressed also in larvae where it is involved in the detection of 2-methylphenol, benzaldehyde, indole, and 3-methylindole. Further functional characterizations of the OR2 orthologs in *C. pipiens* [21] and *A. aegypti* [22] have shown that also in these mosquito species OR2 is narrowly tuned to indole. This structural and functional conservation suggests that OR2 represents an OR sub-family that may play an important role in the life of mosquitoes. Here, we report the identification and characterization of OR2 in *Aedes albopictus* (AalOR2) that represents the first member of the odorant receptor (OR) family of proteins from this mosquito species. As is the case for other members of the OR2 group, AalOR2 shares a great similarity with its orthologs from other mosquito species, and is highly related to its relative in *A. aegypti* (AaeOR2). We show, by using Ca^2+^ imaging in HEK293 cells and the *D. melanogaster* “empty neuron” system, that also AalOR2 responds to a small set of aromatic compounds including indole. Furthermore, AalOR2 expressed in the Drosophila empty neuron is inhibited by (–)-menthone. In agreement with these results, indole and (–)-menthone elicit an attractant and an avoidance effect, respectively, on groups of *A.albopictus* larvae.

## Results

### AalOR2 cloning

**Figure 1:**
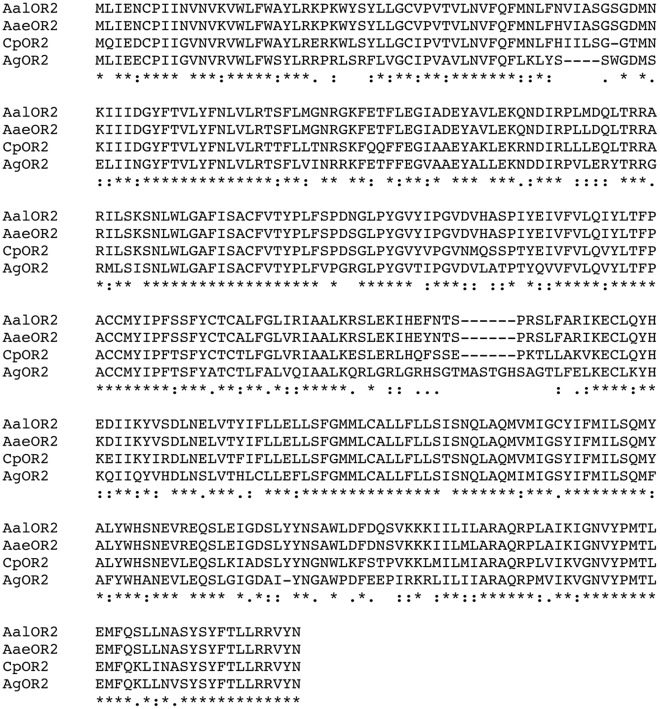
Alignment of mosquito OR2 amino acid sequences. The multiple alignments and calculations of sequence identities and similarities were made using ClustalX (http://www.ebi.ac.uk/Tools/msa/clustalw2/) and protein Blast (http://blast.ncbi.nlm.nih.gov/Blast.cgi), respectively. Clustal notes amino acid alignments as follows: an asterisk indicates a conserved amino acid, a single dot indicates that either the size or hydropathy is conserved, and stacked dots (:) indicate that both the size and hydropathy are conserved. The GenBank accession numbers are JN400274 and JN400275 for the AalOR2 cDNA and genomic sequences, respectively.

In order to clone OR2 in *Aedes albopictus* we carried out RT-PCR experiments with a degenerate pair of primers designed on a multiple sequence alignment of the OR2 orthologs from *Anopheles gambiae*, *Aedes aegypti*, and *Culex pipiens* (see primers section). These primers were used to amplify a partial sequence of AalOR2 from cDNA prepared from manually dissected *A. albopictus* adult antennae. As confirmed by sequencing, we obtained a 651 bp fragment that shared a high degree of homology with its orthologs. Based on this clone, we designed additional gene-specific primers to perform RACE reactions to yield both 5′ and 3′ end AalOR2 sequences (see primers section). Finally, the amplicon obtained, 1.131 bp long, encoded a hyphothetical 376 amino acid polypeptide that was aligned with those of other OR2 sequences from *A. gambiae*, *A. aegypti*, and *C. pipiens*. Literature data [15], [21] show that OR2 is one of the most conserved ORs in these three mosquito species, approaching >80% identity. AalOR2 shares a 71% identity with *A.gambiae,* an 80% identity with *C.pipiens* and a 97% identity with its most evolutionarily related ortholog from *A. aegypti* AaeOR2 (Fig. 1). This result confirms that OR2 is highly conserved in mosquitoes, unlike other ORs for which the sequences are highly divergent. In order to obtain information on the genomic organization we performed a PCR reaction by using two external primers (5′AalOR2Fw/3′AalOR2Rv, see primer section) on genomic DNA. The amplification of a genomic clone, 1.556 bp in length, revealed that the region corresponding to the CDS of the AalOR2 gene shares a genomic organization with the corresponding region of the AaeOR2 gene. Both contain highly conserved 6 exons that are separated by 5 introns, conserved in length and in position. All exon-intron junctional sequences conform to the canonical GT-AG rule.

### RNA Expression

**Figure 2:**
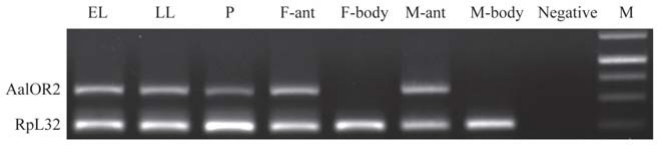
Expression profile of the AalOR2 transcript in Aedes albopictus by RT-PCR. A non-quantitative RT-PCR on enriched poly(A)^+^ RNA showing the expression of AalOR2 mRNA in pre-adult stages, and in antennae from both male and female adult mosquitoes. The lanes are as follows: early larvae (EL), late larvae (LL), pupae (P), adult female antennae (F-ant), adult female carcassae (F-body), adult male antennae (M-ant), adult male carcassae (M-body), negative control obtained by using reaction mix with no cDNA (negative), 1 kb DNA marker-Fermentas (M). All RT-PCR reactions were performed with 40 cycles of amplification on cDNA, except for m-Ant, which was obtained with 35 cycles of amplification on the first-round specific amplificate. Prior to the RT-PCR, the cDNA populations were roughly normalized by RT-PCR to the amount of the AalRpL26 expression levels to ensure an equal input.

In *A. aegypti* and *A. gambiae* it has been reported that OR2 is expressed both in the larval and adult antennae [15], [20]. We examined the expression pattern of the AalOR2 transcript by RT-PCR analyses of tissues isolated from larval and adult stages. In order to check for false amplification from genomic DNA contamination, we used an intro-spanning primer set (3′RACE1Fw/5′RACE1Rev, see primers section) that allowed us to recognize products from either cDNA 624 bp or genomic DNA 889 bp templates. As expected, AalOR2 mRNA is readily detectable in the larval heads throughout all developmental stages, and in the antennae from both male and female adult mosquitoes (Fig. 2). Because one round of PCR was not sufficient to obtain enough amplificate in nanogram amounts starting from the cDNA from the male antennae, we carried out a second round of PCR with an aliquot of the first round products using the same gene-specific primers. Although we performed a nonquantitative PCR, our data seem to suggest that in *A. Albopictus* the *AalOR2* gene is more expressed in the female antennae than in the male antennae, as previously suggested for the *AgOR2* gene [23] (Fig. 2). In these experiments we used AalRpL26 as an internal control to verify the quality of the cDNA.

### Study of the Odorant Response Profile of AalOR2: “in vitro” and “in vivo” Approaches

Recently, AgOR2, AaeOR2 and CpOR2 have been shown to be strongly tuned to indole [18], [21], [22]. In order to establish the response profile of AalOR2, we used an “in vitro” approach, performing Ca^2+^ imaging in a heterologous cell system and an “in vivo” experiment, using single sensillum recordings (SSR) in an engineered neuron of *?-halo Drosophila melanogaster* expressing AalOR2 in the empty neuron.

### Ca^2+^ Imaging Measurement with Fura2/AM in HEK293 Cells

**Figure 3:**
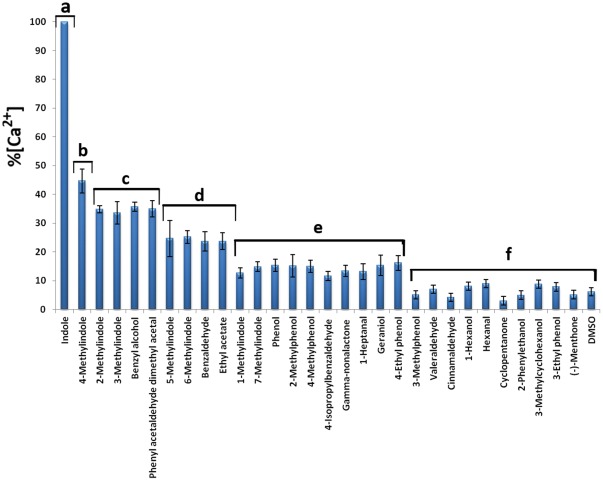
Quantification of the intracellular Calcium ([Ca^2+^]i) in HEK293 cells expressing AalOR2 along with DmOR83b, in response to the compounds reported in Tab.1. A free intracellular Ca^2+^ concentration was determined using the 340/380 excitation ratio for Fura-2. Each response was normalized to the maximum responder. AalOR2 is strongly tuned to indole, and responds with a lower sensitivity to other methylindoles, and benzaldhyde. Furthermore, benzyl alcohol and phenyl acetaldehyde yielded responses that were at least 35% of the response to indole. The results shown are the means of five separate experiments (n = 5). Error bars =  S.E.M. A 1-way ANOVA test (df among groups = 30, df within group = 124, F-ratio = 214,44, p≤0,05) was performed followed by a Bonferroni correction for multiple test comparisons considering as significant the modified Bonferroni p≤1,67×10^−3^. Letters on top of the bars indicate that means differ significantly from one another.

In order to obtain an in vitro expression in HEK293 cells (Human Embryonic Kidney 293 cells), the AalOR2 coding sequence was cloned into the pHM6 vector. The HEK293 cells were transiently co-transfected with AalOR2 and the co-receptor Orco from *D. melanogaster* [12], [13]. These transfected cells were tested against a panel of 30 odorants as reported in Table 1, including some chemicals known to activate mosquito OR2, that allowed us to make a functional comparison of AalOR2 with its orthologs. Each odor was applied into the dish containing the cells to a final concentration of 10^−6^ M, producing a calcium response, that was negligible in the cells exposed to the solvent alone (Fig. 3). The free intracellular Ca^2+^ concentration was determined using a 340/380 excitation ratio for Fura-2, and fluorescence images were acquired using a cooled CCD camera controlled by TILL Vision software. As already reported for AgOR2, CpOR2 and AaeOR2 [18], [21], [22], AalOR2 responded to a set of aromatic compounds, such as indole, each of the methylindoles and benzaldehyde. Each of these odors activated the receptor to different levels as detected by different variations in fluorescent intensity, with indole eliciting the highest increase of the intracellular Ca^2+^ concentration (Fig. 3). Furthermore, benzyl alcohol and phenyl acetaldehyde yielded responses that were at least 35% of the response to indole. Each odorant was assayed in five plates, each in triplicate; the control HEK-293 cells were indifferent to any odor tested (data not shown).

**Table 1:** Table of compounds used in the Single Sensillum Recordings and Ca^2+^ imaging assays in heterologous cells.

COMPOUNDS	CLASSIFICATION
INDOLE	INDOLE
1-METHYLINDOLE	INDOLE
2-METHYLINDOLE	INDOLE
3-METHYLINDOLE	INDOLE
4-METHYLINDOLE	INDOLE
5-METHYLINDOLE	INDOLE
6-METHYLINDOLE	INDOLE
7-METHYLINDOLE	INDOLE
PHENOL	PHENOL
2-METHYLPHENOL	PHENOL
2-METHYLPHENOL	PHENOL
4-METHYLPHENOL	PHENOL
3-ETHYLPHENOL	PHENOL
4-ETHYLPHENOL	PHENOL
BENZALDEHYDE	ALDEHYDE
CINNAMALDEHYDE	ALDEHYDE
GAMMA NONALACTONE	ALDEHYDE
PHENYL ACETALDEHYDE DIMETHYL ACETAL	ALDEHYDE
4-ISOPROPYL BENZALDEHYDE	ALDEHYDE
HEXANAL	ALDEHYDE
1-HEPTANAL	ALDEHYDE
VALERALDEHYDE	ALCOHOL
2-PHENYLETHANOL	ALCOHOL
GERANIOL	ALCOHOL
1-HEXANOL	ALCOHOL
BENZYLALCOHOL	ALCOHOL
3-METHYLCYCLOEXANOL (3MCE)	ALCOHOL
CYCLOPENTANONE	KETONE
(–)-MENTHONE	KETONE
ETHYLACETATE	ESTERE

### Single Sensillum Recording (SSR) of AalOR2 Expressed in the Delta Halo Drosophila Mutant

In our studies *in vivo,* the AalOR2 coding region was cloned downstream of the UAS sequences in pUAST [24], and activated by GAL4 under the *OR-22a* promoter, which is expressed only in the ab3A neuron. UAS(AalOR2) was expressed in the Delta-halo *Drosophila* mutant [25], [26], that lacks its endogenous Odorant Receptor genes, *OR22a* and *OR22b*. In our experiment, we used also UAS(mCD8-GFP), that localizes GFP to the cell membranes as it encodes the mouse lymphocyte surface marker CD8 fused in frame with the GFP protein. When activated by GAL4, the specifically GFP-labeled sensilla are visible in live flies, thereby allowing them to be distinguished and recorded electrophysiologically. The AalOR2, expressed in this “empty neuron”, was challenged with the same odor panel as that used with the HEK293 cells (Tab.1), and its activity was measured in terms of action potential frequency. We used a pulse duration of the olfactory stimulus of 0.5 s (seconds). Recordings of the action potentials were collected using AUTOSPIKE (Syntech) and analyzed by custom spike-sorting software. In our current study, all compounds were administered as 10^−3^ M dilutions.

**Figure 4:**
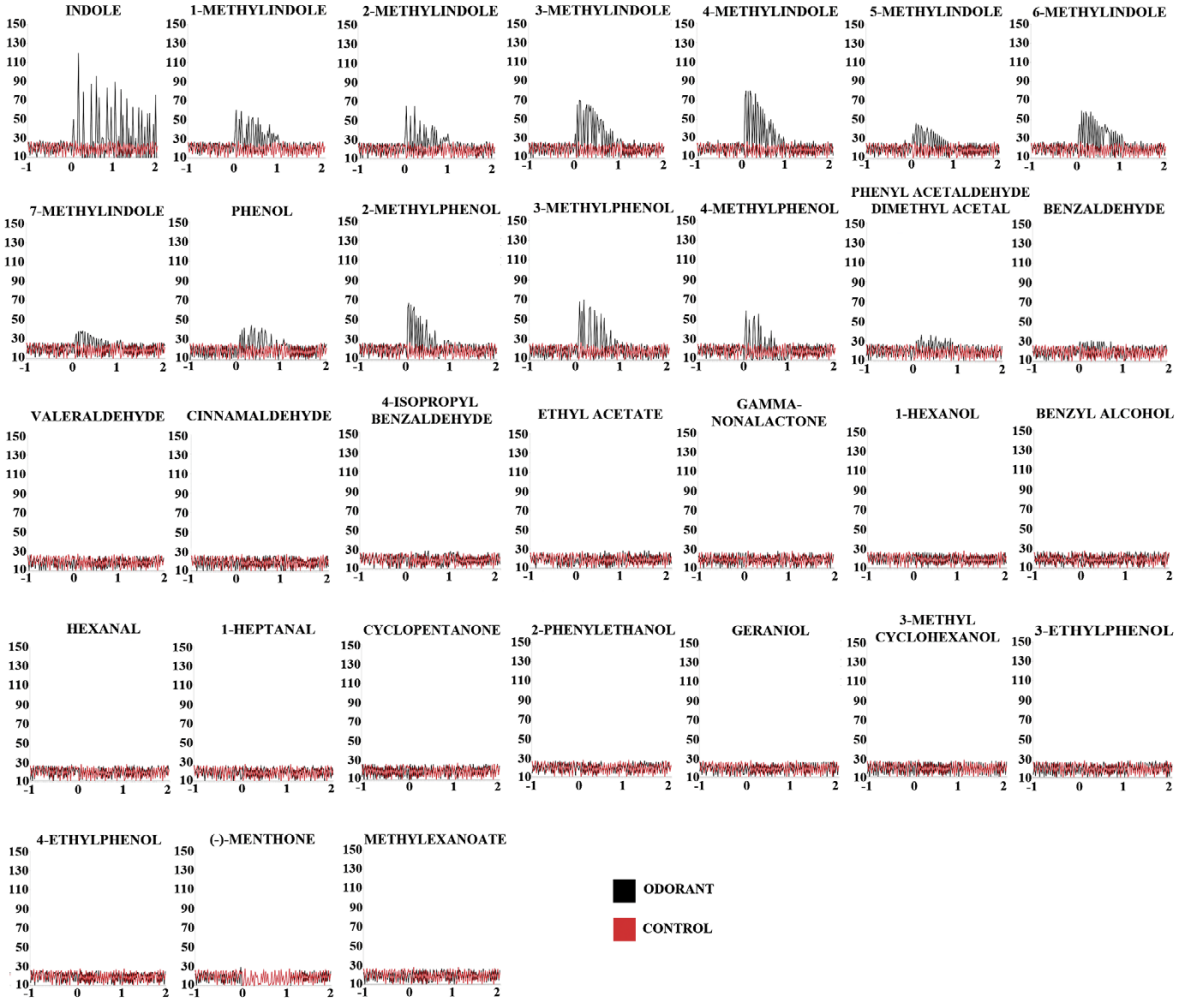
Peristimulus-time histograms. Each panel reports a peristimulus-time histogram illustrating the temporal dynamics of the responses to each odor of table1. In each panel, point 0 indicates the onset of the 0.5 sec odorant stimulus. The olfactory responses were assayed as firing rates in consecutive 50 ms intervals beginning 1 sec before the odor stimulus and continuing for another 1 sec. Each panel is representative of just one AalOR2 OSN.

**Figure 5:**
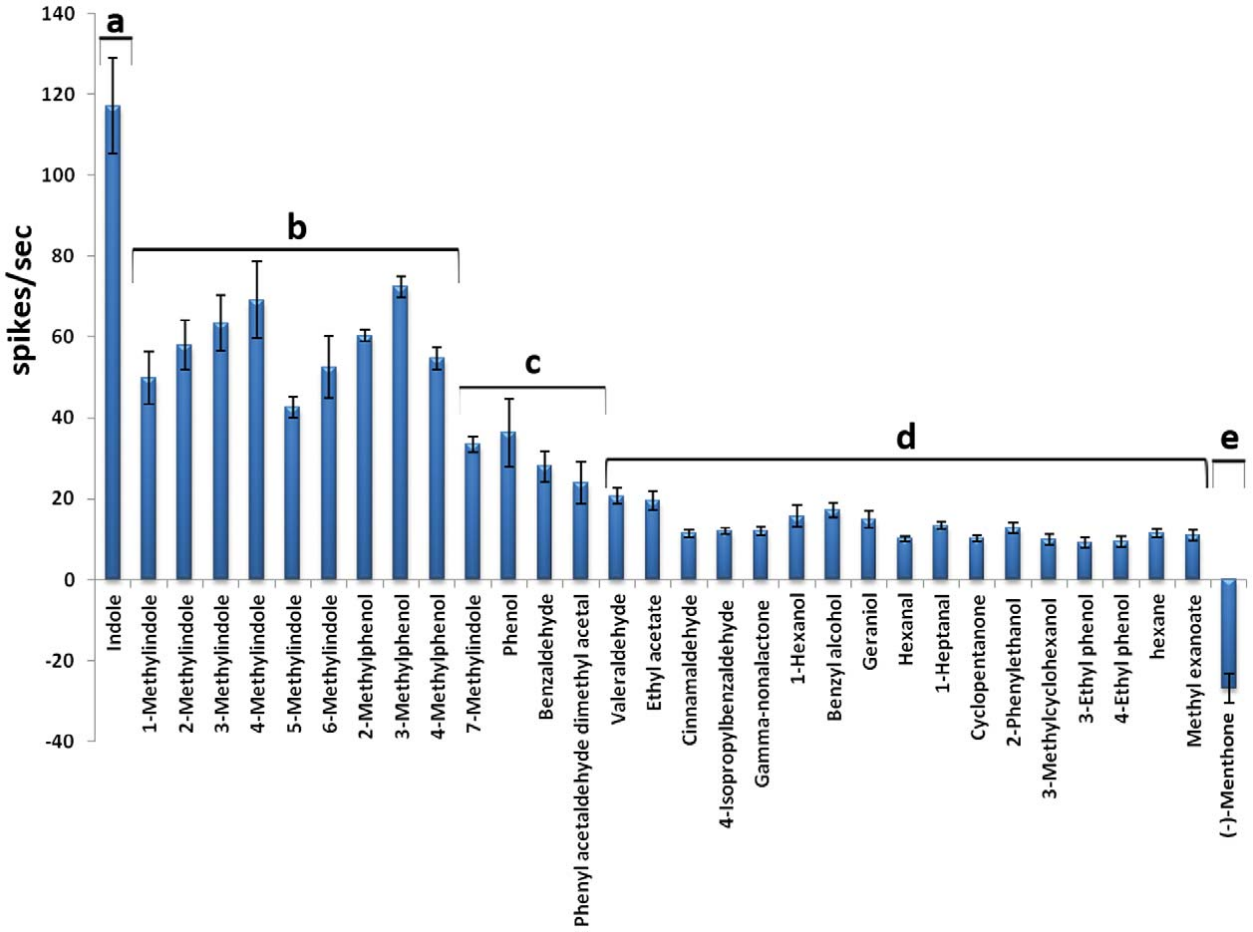
Single Sensillum Recording assay. The ab3A neuron in the Delta-halo mutant expressing UAS(AalOR2) was challenged with the panel of compounds reported in Tab.1. Each odor was applied at a concentration of 10^−3^ M with a pulse duration of 0.5 sec. Stimuli were used for a maximum of 3 presentations. The responses were quantified by subtracting the number of impulses in 1 sec of unstimulated activity from the number of impulses in the 1 sec following odor stimulation. A maximum of three sensilla were analyzed per fly, 4–10 days old, for a total of three flies per each transgenic strain. (mean ± S.E.M., n = 3). It is clearly evident that AalOR2 interacts with some aromatic compounds, although indole triggered a narrow strong activation. A 1-way ANOVA test (df among groups = 30, df within group = 248, F-ratio = 51,57, p≤0,05) was performed followed by a Bonferroni correction for multiple test comparisons considering as significant the modified Bonferroni p≤1,67×10^−3^. Letters on top of the bars indicate statistical significant changes.

**Figure 6:**
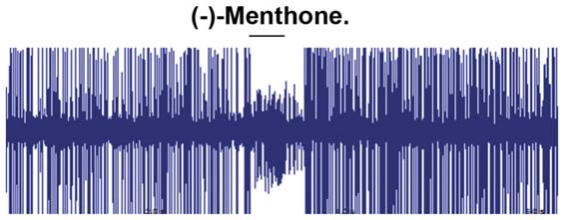
Inhibitory effect of (–)Menthone on the AalOR2-“empty-neuron”. Representative trace of Single-Sensillum Recordings from AalOR2-ORCo in the ab3A ORN, in response to 10^−3^ M (–)-Menthone. The firing rates clearly indicate a decrease in the number of large spikes corresponding to the ab3A neuron in one second of activity. Like with the other odors, the experiments were carried out testing three independent UAST insertions and identical results were observed in all the lines. Three sensilla were analyzed per fly, for a total of three flies per each transgenic strain.

To be certain that the endogenous odorant receptor in the empty neuron of our transgenic flies was lacking, we used ethyl butyrate [25], that elicits a strong excitatory response from the ab3A neuron only in the presence of OR22a. As expected, this chemical failed to elicit a response (data not shown). In order to obtain a direct indication of the response magnitude and kinetics, we generated peri-stimulus time histograms reporting the firing frequencies of AalOR2 as a function of time (Fig. 4). Firing rates were quantified by the number of spikes elicited in one second after stimulation compared to the spontaneous activity of the neuron (Fig. 5). We found that at a 10^−3^ M concentration, indole elicited the strongest response from AalOR2, inducing more than 130 spikes/sec. Moreover, as already reported for the *in vitro* assay, AalOR2 responded to several aromatic compounds such as each of the methylindoles and methylphenol. Most of the other odorants tested in our experiments elicited virtually no responses from AalOR2 (Fig. 5). Interestingly, when we used synthetic (–)-menthone (2*S*, 5*R*-*trans*-2-isopropyl-5-methylcyclohexanone), down to a 10^−3^ M concentration, we observed a strong inhibition of neuronal activity (Fig. 6).

**Figure 7:**
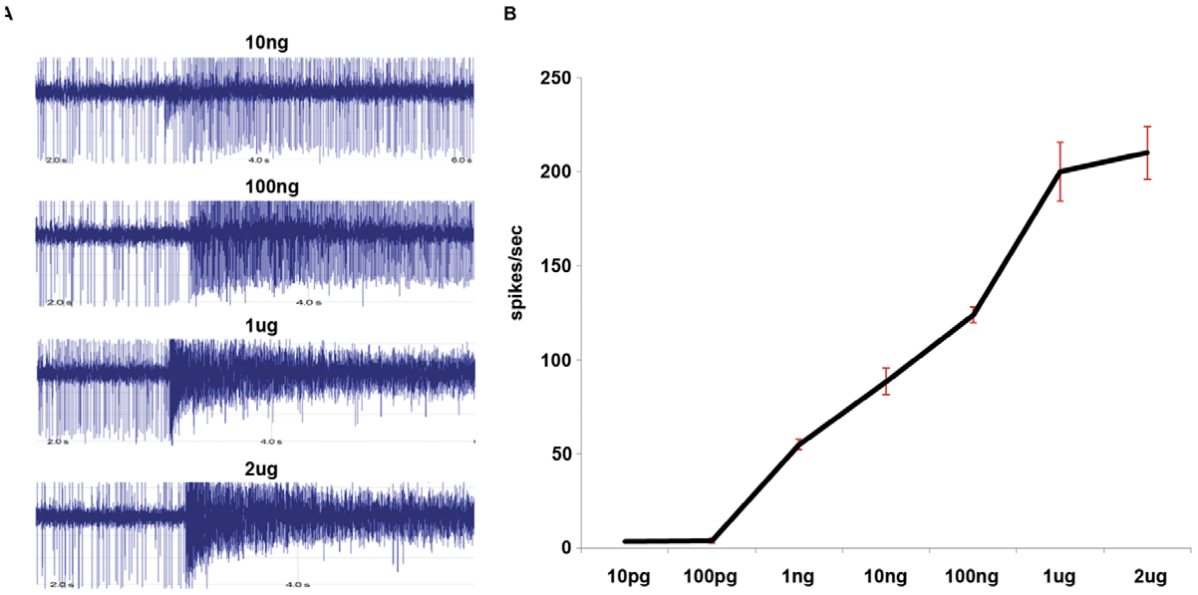
Dose dependent response of AalOR2 to indole. A) The firing rates of the AalOR2-“empty neuron” in response to increasing concentrations of indole ranging from 10^−7^ M to 2 ×10^−2^ M. Indole led to an increase in spike frequency directly proportional to its concentration, suggesting its specificity of action. B) The same responses as in A) are reported in a graph. The red bars represent the means ± S.E.M. of three separate experiments (three sensilla per fly; n = 3).

To confirm that indole was a specific agonist of AalOR2 we performed an indole dose-response experiment, with a concentration range from 10^−5^ M to 2 ×10^−2^ M. Our results clearly indicated that the neuronal spike frequency was directly proportional to the concentration of indole, as shown in Fig. 7A,B. In summary, our results show that AalOR2 is strongly excited by indole and to a similar degree inhibited by (–)-menthon which is a novel odorant able to elicit an inhibitory response from AalOR2.

### Indole and (–)-Menthone Elicited Behavioral Responses in A.albopictus Larvae Groups

**Figure 8:**
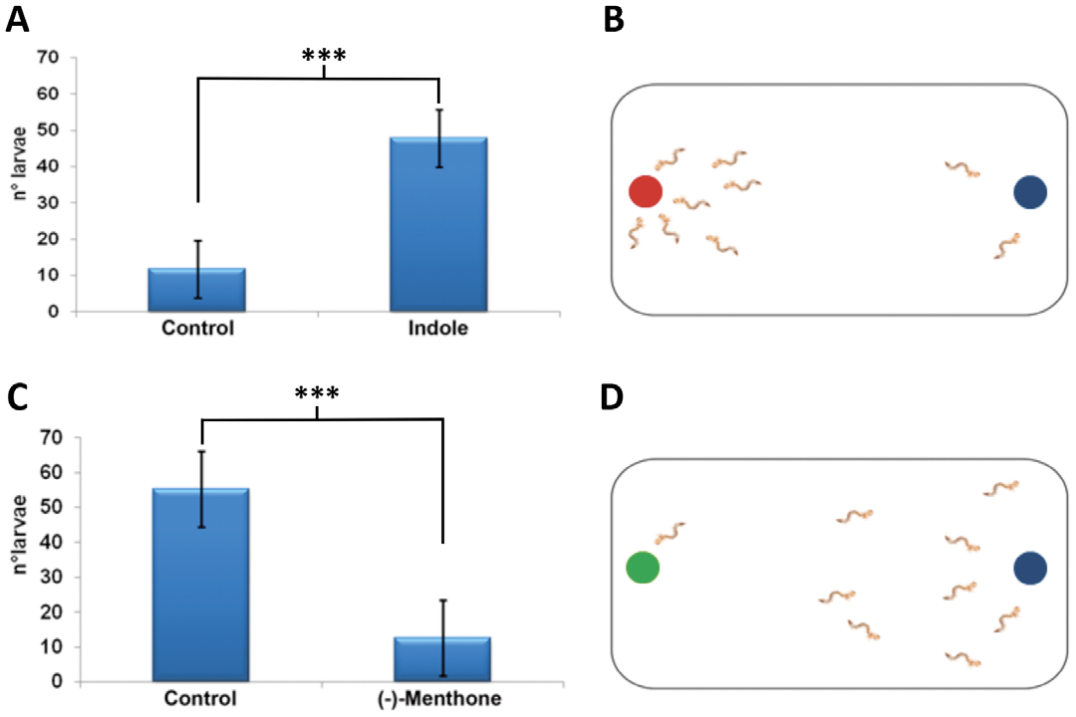
Behavioral assay on *A.albopictus* larvae. (A, B) Third and fourth instar larvae were kept in a pirex pan containing a ball of low melting agarose with 10 mM indole and a ball of low melting agarose (the red and blue spots, respectively, in B). The number of larvae were counted each 30 seconds for a maximum of 22 minutes. The histogram shows clearly that indole has an attractive effect for *Aedes albopictus* larvae. (C,D) Third and fourth instar larvae in the presence of a ball containing 10 mM (–)-menthone and a ball of low melting agarose (the green and blue spots, respectively, in D). Again, the larvae were counted each 30 seconds for a maximum of 22 minutes. Histogram C shows clearly that (–)-menthone evoked an avoidance behavior on the larvae. The result was calculated reporting the number of larvae just at a discrete15-min time-point. These values were compared with each other and analyzed for statistical significance by using a paired, two-tailed student’s t test considering p≤0.05 as significant (df = 4 and t_0,05;4_ = 44,28 for behavioral test A; df = 4 and t_0,05;4_ = 27,76 for behavioral test C. *** indicate statistical significant changes (two-tailed Student’s t-test, p<0.001). Error bars indicate S.E.M. for n = 5 separate trials per odorant.

The olfactory molecules already play an important role for mosquitoes in the early larval stages. Previous works have shown that *A.gambiae* larvae respond to olfactory stimuli with specific behavioral responses that are mediated by the larval olfactory system [20], [27]. Since in *A.albopictus*, AalOR2 is expressed in the larval heads, as well as in the adult antennae, we decided to assay the odorant-specific responses of *A.albopictus* larvae groups. To achieve this aim, we used the simple mobility paradigm developed by Xia et al. [20] to assay the behavioral responses of *A. albopictus* larvae to indole and (–)-menthone, two odorants that evoked strong responses of AalOR2 in our SSR experiment. In our test, we used a videocamera to record the behavior of large groups of late instar larvae in response to a 10^−2^ M source dilution of indole and (–)-menthone. In our experimental arena we defined a test zone and a control zone each corresponding approximately to ¼ of the total area around the odor balls. The number of larvae in both the test and control zones were counted every 30 s over a 22-min assay. Although this end-point analysis does not allow a detailed characterization of the attractive and repulsive behavioral patterns, our data nevertheless provide preliminary evidence of larval olfactory-based behaviors to indole and (–)-menthone. The larvae responded strongly to indole, with about 54 larvae approaching the indole zone, at a discrete 15 min time point; the number of larvae near the indole zone decreased towards the end of the experiment. This behavior could be consistent with the effect of an attractant. Conversely, (–)-menthone elicited an avoidance behavior on larvae, with just a few of them approaching the menthone zone (Fig. 8A,B); moreover, although we did not measure the parameters associated with the directional movement of the larvae, it appeared clear that in response to (–)-menthone the larvae that approached the menthone zone displayed an increase in terms of overall movement and velocity, suggesting a repulsive effect.

### **Discussion**

Here we report the identification and characterization of AalOR2, that represents the first member of the OR family of proteins from the mosquito *A. Albopictus* to be reported. To date, orthologs of this receptor have been reported for *A.gambiae*, *A.aegypti* and *C.pipiens* [18], [22], [21]. Despite the fact that insect ORs display a high degree of divergence, the three OR2 orthologs characterized to date share an average of 80% amino acid identity. As expected, AalOR2 also shares an extremely high proportion of its primary amino acid sequence with other OR2 sub-family members from other mosquito species [21], [22], with an identity of 97% shared with AaeOR2.

Last year, several authors [22], [21] suggested that this sequence homology was strictly correlated to odorant specificity. In fact, it has been reported that the highly conserved AgOR2, AaeOR2 and CpOR2 orthologs share a similar narrow response to indole. On the basis of these results Bohbot and collaborators [22] hypothesized that this high sensitivity to indole could represent an ancient ecological adaption preserved due to its importance in some aspects of the mosquito life cycle. However, the same authors also reported the identification of OR2 orthologs from additional zoophilic and anthropophilic mosquito species, suggesting that the role of the mosquito OR2 does not seem to be strictly associated with host selection. AalOR2 is expressed in the antennae of both the larvae and adult mosquitoes, in which it seems to have a female-biased expression, which is in agreement with the expression pattern of its mosquito orthologs from *A. gambiae* and *A. aegypti* [20], [15]. Its expression in the larval stage suggests an important role of this gene in larval feeding and/or other behaviors, while in adults it could have an important role for females in searching for suitable oviposition places. Like the other OR2 orthologs, AalOR2 is also narrowly tuned to indole, as detected by its expression in mammalian cells and in the *D. melanogaster* “empty neuron”. In both our expression systems, we used the *D. melanogaster* Orco as heterodimerization partner; our data show that, as for the other OR2 ortologs functionally characterized to date, AalOR2 responds with a lower sensitivity to other methylindoles, 2methylphenol and benzaldhyde, thus further confirming a structural and funtional conservation of the mosquito OR2 orthologs. Indole, which constitutes nearly 30% of the volatile headspace of human sweat [19], is a ubiquitous volatile compound that has been linked to host seeking, and oviposition in aedine [28], [29] as well as in anopheline mosquitoes [30].

In our SSR assay (–)-menthone produced an inhibitory effect on the AalOR2 expressed in the “empty neuron” of *D. melanogaster*, in the absence of applied odorants. We also showed, through a larval behavioral assay, that indole and (–)-menthone, offered to *A.albopictus* larvae, evoked an attractive and avoidance effect, respectively. It has already been demonstrated that indole is an attractive compound for *A.gambiae* larvae and that other molecules, like DEET, can have a repellent effect [20]. (–)-menthone is an organic compound belonging to the ketone family and is a component of some essential oils such as *Mentha microphilla* that have insecticidal properties [31]. The inhibition of the odorant receptors by the odorant molecules has been widely reported; for example, Carlson and colleagues found that 6-MHO, that is a fly repellent produced by cows, inhibited AgOR1 and activated AgOR21 [18]. It is now well accepted that certain odorants activate some receptors but inhibit others, indicating odorant information can be integrated already at the antennal level. More studies aimed at increasing our understanding of the way (–)-menthone acts in the presence and absence of other odorants, such as the activating chemicals, its activity on other OR2 orthologs and its function on other characterized ORs are planned for the near future.

## Materials and Methods

### Mosquito Rearing and Blood Feeding

*Aedes albopictus* (Napoli strain) embryos were generated in-house and disinfected with 0,05% sodium hypochlorite prior to hatching in flat plastic pans with distilled water. Larvae were reared on a diet of ground Whiskas Original Recipe cat food (Kalkan Inc. USA) that was applied to the surface of the water. The pupae were transferred to plastic cups in one-deciliter plastic containers, where the newly emerged adults were collected the following morning. The adult mosquitoes were maintained in one-deciliter plastic containers at 27°C with 75% relative humidity under a 12∶12 h photoperiod and provided with a 10% destrose solution. The 3-4-day-old adult females were blood-fed on human volunteers using standard protocols.

### Identification, Cloning and Expression Pattern of AalOR2

The degenerate primers (see primers section) were designed using the better conserved region on the aligned protein sequences of the *A.gambiae*, *A.aegypti* and *C.pipiens* OR2 orthologs, obtained from the http://www.vectorbase.org/database. The molecular techniques were carried out according to the general protocol reported in Sambrook [32]. The adult antennae and larval heads were manually dissected from animals anesthetized with ether, immediately frozen in dry ice and subsequently processed. The total RNA was extracted by using TRI Reagent (Sigma) according to the manufacture’s instructions. The enriched poly(A)+ RNA was prepared using the QuickPrep Micro mRNA Purification Kit (GE Healthcare), following the manufacture’s instructions. 2 µl of RNA were loaded on 1% agarose gel and quantized using an RNA marker (Fermentas) as reference, through the Gene tools software (Perkin Elmer). 0.3 to 1 µg of RNA was retro-transcribed into the cDNA using the enzyme Reverse Transcriptase (Fermentas) and 500 ng of anchor primer-dT (see primers section). The PCR reactions with degenerate primers were carried out in a 50 µl final volume containing 0.2 mM deoxynucleotides (Fermentas), 1 unit of Phusion High-Fidelity DNA Polymerase (Finnzymes) and 2.5 mM primers in the correspondent buffers. Two percent of the synthesized cDNA was amplified by PCR with degenerate primers. All the amplicons were analysed by electrophoresis in agarose gels. The amplified fragments were cloned using the pGEMT-easy cloning vector (Promega) following the manufacturer’s instructions, and sequenced at PRIMM Biotech.

In order to study the expression pattern, tissue dissection, RNA extraction and cDNA synthesis were performed as described above, starting from 50 antennae pairs and 50 larval heads. The PCR reactions were carried out using equivalent amount of cDNA in a final volume of 25 µl. The integrity of each cDNA template was confirmed by the amplification of a ribosomal AalRpL26 protein encoding transcript. PCR amplification products were run on a 1.5% agarose gel and verified by DNA sequencing.

The multiple alignments and calculations of sequence identities and similarities were made using ClustalX (http://www.ebi.ac.uk/Tools/msa/clustalw2/) and pBlast (http://blast.ncbi.nlm.nih.gov/Blast.cgi). The GenBank accession numbers are JN400274 and JN400275 for the AalOR2 cDNA and genomic sequences, respectively.

### Primers Section

#### Degenerate primers

AalOR2Fw1: TGGYTNTTYTGGWSNTAYYT.

AalOR2Fw2: GGNTAYTTACNGTNYTNTAYTT.

AalOR2Rev1: TGRAACATYTCNARNGTCAT.

AalOR2Rev2: CATRAADATRTANSWNCCDATCAT.

#### 3'RACE primers

3′RACE 1Fw: TTCGGACGTCGTTCCTAATG.

3′RACE 2Fw: GCAAGGATTCTGTCCAAGTCGA.

3′RACE 3Fw: GCGTCGCCAATTTACGAAATTG.

3′RACE 4Fw: GAGTGTCTCCAATATCACGAGG.

3′RACE 5Fw: GTTGAGCATCAGCAATCAGCTG.

#### 5'RACE primers

5′RACE 1Rv: AGCAGCCGATCATTACCATCTG.

5′RACE 2Rv: TTATCCTCGCAAATAGCGACCG.

5′RACE 3Rv: CTGCAGCACGAACACAATTTCG.

5′RACE 4Rv: CAGAGGATAGGTCACGAAGCAA.

5′RACE 5Rv: TTCGAACTTGCCTCGGTTTCCCAT.

#### anchor primer-dT

GACCACGCGTATCGATGTCGACTTTTTTTTTTTTTTTT.

#### 5' Fw primers and 3' rev primers of aedes albopictus OR2

5′AalOR2Fw: ATGTTGATAGAAAATTGTCCA.

3′AalOR2Rv: TTAATTATAAACTCTCCGAAGC.

### Cloning of the Genomic Sequence of AalOR2

Genomic DNA was prepared starting from about ten *A. albopictus* larvae through the Mammalian Genomic DNA Miniprep Kit (Sigma) following the manufacture’s instructions. About 100ng was used as template in a PCR reaction with the primer combination 5′AaOR2Fw/3′AalOR2Rev (see primers section).

### Calcium Imaging Assay Through Fura2/AM in HEK293 Cells

The full-length CDS of AalOR2 was amplified with 5′AalOR2Fw and 3′AalOR2Rv primers (see primers section) and cloned in pHM6 mammalian expression vector. 5×10^5^ HEK293 cells (Human Embryonic Kidney 293 cells) were seeded in a single dish. After 24 hours, the cells were transiently co-transfected with pHM6/AalOR2 along with pHM6/*D.melanogaster* Orco. The reagent used for this transfection was Roti Fect Plus (Carl Roth), and the ratio used between Roti Fect Plus and DNA was 5∶1. The amount of DNA used was 1µg for each Odorant Receptor. 48 hours after transfection, 2µM Fura2/acetomethylester (Invitrogen) were loaded in the dish containing the cells and incubated for 20 minutes in the dark, prior to each assay. After this time, the medium containing the Fura2 calcium dye was removed, and 2mL of SES solution (Standard External Solution containing -in mM- 135 NaCl, 5 KCl, 1 CaCl2, 1 MgCl2, 10 HEPES and 10 glucose, pH7.4) was added to the cells. All the odorants used were >99% pure or of the highest grade commercially available. These chemicals were dissolved in DMSO and applied in the dish using a microsyringe to a final concentration of 10^−6 ^M and baseline readings were taken for 20 sec before the addition of odorant. The fluorescence images were acquired using a cooled CCD camera controlled by TILL Vision software, and the free intracellular Ca^2+^ concentration ([Ca^2+^]) was determined by using the fluorescence ratio method (340/380), with background subtraction. The fluorescence images were taken at a frequency of 1/5 s for the duration of each assay. The responses were quantified by the ratio of the maximum fluorescent reading of a cell divided by the baseline reading of that cell prior to odorant addition. (maximum/minimum response), and each response was normalized to the maximum responder that in our case was indole. Each odor was assayed in triplicate per plate for a total of five plates. Statistical analysis of differences in the normalized responses was carried out by a one-way ANOVA test with a Bonferroni correction.

### Drosophila Melanogaster Stocks

Drosophila strains were maintained on standard food. The flies used in our experiments were the following:

Δhalo strain (kindly provided by John R.Carlson Yale University): w; Δhalo/CyO;Dr/TM3,Sb.

UAS-mCD8-GFP strain (Drosophila Stock Center Bloomington, IN): P{w[^+^mC] = UAS-mCD8::GFP.L}LL4, y[1] w[*]; Pin[Yt]/CyO;

Or22a-Gal4 strain (Drosophila Stock Center -Bloomington, IN): w[*]; P{w[+mC] = Or22a-GAL4.7.717}14.2.

UAS-AalOR2 strain: w; Δhalo/If; TM3,Sb/UAS-AalOR2.

To obtain the UAS-AalOR2 transgenic strain, the full-length CDS of AalOR2 was cloned into the pUAST vector (24), in frame with the Cavener sequence. This plasmid was extracted with Plasmid Midi Kit (Qiagen) and injected into the w^1118^ strain by Genetic Services (http://www.geneticservices.com/injectionservices.htm). The UAS-AalOR2 transgenic strain was finally crossed with the P{w[^+^mC] = UAS-mCD8::GFP.L}LL4, y[1] w[*];Δhalo/CyO; P{w[+mC] = Or22a-GAL4.7.717}14.2 strain in order to obtain the final strain P{w[+mC] = UAS-mCD8::GFP.L}LL4, y[1] w[*];Δhalo/Δhalo; P{w[+mC] = Or22a-GAL4.7.717}14.2/UAS-AalOR2, that was subsequently used.

### Electrophysiological Recordings (Single Sensillum Recordings)

Single sensillum recordings were carried out essentially according to Clyne, 1997 and Stensmyr, 2003 [33], [34]. A 5- to 10-day-old fly expressing AalOR2, was mounted in a truncated pipette tip with the antenna protruding from the narrow end. The pipette tip was fixed with wax on a microscope slide, and the antenna gently placed on a cover-slip and stabilized with a glass electrode. The antennal surface was observed at a 1000x magnification, which allowed the individual sensilla to be clearly resolved, through an Olympus BX51 microscope fitted with fluorescence optics to view GFP. The action potentials of the ORNs in the sensillum were recorded by placing an electrode through the sensillum wall into contact with the lymph that bathes the dendrites. For the recording electrode, a glass capillary with the tip drawn to 1µm diameter was filled with sensillum lymph ringer and slipped over an AgCl-coated silver wire. The indifferent electrode was filled with Ephrussi and Beadle solution and was put into the eye. The extracellular analog signals originating from the OSNs were amplified 1000 times, digitally converted via Syntech IDAC-4 USB and visualized by Syntech Autospike 3.2. The signal was also fed to a loudspeaker for audio monitoring. The recordings of the action potential were stored on the PC and all analysis was carried out with the AUTOSPIKE software. The separation of the activity of the collocated ORNs in the single sensillum was based on differences in spike amplitude. The ORN with the largest spike amplitude corresponded to neuron A. The odor stimulation duration was 0.5 s [33]. The chemicals were >99% pure or of the highest purity available and were diluted (v/v) in redistilled hexane down to a concentration of 10^−3^ M. The odorant stimuli were prepared in Pasteur pipettes and were presented by placing the tip of the pipette through a hole in a tube carrying a purified air stream (50 ml/s) directed at the fly and by administering a pulse of charcoal-filtered air (0.5 m/s) through the pipette containing the odorant [33]. The stimuli were used for a maximum of three presentations. The responses were quantified by subtracting the number of impulses in 1 sec of unstimulated activity from the number of impulses in the 1 sec following odorant stimulation. For each odorant, each recording was obtained from a separate sensillum, with no more than three sensilla analyzed per fly. Three flies for three independent UAST insertions were analyzed. Statistical analysis of differences in the responses was carried out by a one-way ANOVA test with a Bonferroni correction.

### Larval Behavior Assay and Data Analysis

An olfactory-based behavioral assay for groups of *Aedes albopictus* larvae was carried out by using a larval behavior assay as described in ref. 20. One hundred *Aedes albopictus* third and fourth instar larvae were picked and washed carefully. The washed larvae were kept in 27°C distilled water and starved for 2 h. Specific amounts of (–)-menthone and indole ([]_f_ = 10 mM) were dissolved in preheated 2% agarose low melting. The test was performed in a 25×38×5 cm pyrex pan containing 1000 ml of 27°C distilled water.

A test zone and control zone, corresponding to a region around the agarose balls, equal to ¼ of the total area, were determined. The odorant/control agarose balls were placed in the center of the zone area, using a mesh ring. The larvae were released in the middle of the plate and allowed to swim freely for 1hr. The larval behavior was recorded by a video camera for 22-minutes. The numbers of larvae in both the test and control zones were counted every 30s, but a performance index (PI) [20] was calculated just at a discrete15-min time-point as follows: PI** = **(#odorant**-**#control)/(#odorant**+**#control), where the # odorant indicates the number of larvae in the test zone and the # control indicates the number in the control zone. Each odorant was assayed in five independent trials.

The PI values (+54 for indole and −80 for (−)-menthone) were compared with each other and analyzed for statistical significance by using a paired, two-tailed student’s t test. A one-way ANOVA test was performed to asses the statistical significance of differences for all comparisons in Fig. 3 and 5, followed by a Bonferroni correction for multiple comparisons.
